# *Neph1* is required for neurite branching and is negatively regulated by the PRRXL1 homeodomain factor in the developing spinal cord dorsal horn

**DOI:** 10.1186/s13064-024-00190-6

**Published:** 2024-07-24

**Authors:** João Baltar, Rafael Mendes Miranda, Maria Cabral, Sandra Rebelo, Florian Grahammer, Tobias B. Huber, Carlos Reguenga, Filipe Almeida Monteiro

**Affiliations:** 1 Unidade de Biologia Experimental, Departamento de Biomedicina, FMUP - Faculdade de Medicina da Universidade do Porto, Porto, Portugal; 2 Pain Neurobiology, IBMC - Instituto de Biologia Celular e Molecular, Porto, Portugal; 3 i3S - Instituto de Investigação e Inovação em Saúde, Universidade do Porto, Porto, Portugal; 4 Departamento de Patologia Clínica, Centro Hospitalar Universitário São João, Porto, Portugal; 5 III. Department of Medicine, University Medical Center Hamburg-Eppendorf, Hamburg, Germany; 6 Hamburg Center for Kidney Health (HCKH), University Medical Center Hamburg-Eppendorf, Hamburg, Germany

**Keywords:** Dorsal root ganglion, Dorsal spinal cord, Mouse development, Transcription factor, Prrxl1, Neph1, ChIP-seq, Sholl analysis

## Abstract

The cell-adhesion molecule NEPH1 is required for maintaining the structural integrity and function of the glomerulus in the kidneys. In the nervous system of *Drosophila* and *C. elegans*, it is involved in synaptogenesis and axon branching, which are essential for establishing functional circuits. In the mammalian nervous system, the expression regulation and function of *Neph1* has barely been explored. In this study, we provide a spatiotemporal characterization of *Neph1* expression in mouse dorsal root ganglia (DRGs) and spinal cord. After the neurogenic phase, *Neph1* is broadly expressed in the DRGs and in their putative targets at the dorsal horn of the spinal cord, comprising both GABAergic and glutamatergic neurons. Interestingly, we found that PRRXL1, a homeodomain transcription factor that is required for proper establishment of the DRG-spinal cord circuit, prevents a premature expression of *Neph1* in the superficial laminae of the dorsal spinal cord at E14.5, but has no regulatory effect on the DRGs or on either structure at E16.5. By chromatin immunoprecipitation analysis of the dorsal spinal cord, we identified four PRRXL1-bound regions within the *Neph1* introns, suggesting that PRRXL1 directly regulates *Neph1* transcription. We also showed that *Neph1* is required for branching, especially at distal neurites. Together, our work showed that Prrxl1 prevents the early expression of *Neph1* in the superficial dorsal horn, suggesting that *Neph1* might function as a downstream effector gene for proper assembly of the DRG-spinal nociceptive circuit.

**Supplementary Information:**

The online version contains supplementary material available at 10.1186/s13064-024-00190-6.

## Introduction

The proper processing of sensory information depends on appropriate connections between peripheral dorsal root ganglion (DRG) afferents and their central targets in the spinal cord. In fact, central terminals of DRG afferent fibers segregate into specific laminae within the dorsal horn of the spinal cord depending on the sensory modality and the peripheral tissues innervated [1]. During development, extrinsic and intrinsic signals are required for the generation of diverse neuronal subtypes and specialization of sensory neurons [2]. Highly coordinated genetic programs, orchestrated by the combinatorial action of distinct transcription factors, instruct several developmental cellular activities, such as neuronal differentiation, migration, axon guidance, branching and synaptogenesis [3–6]. Although several advances have been made in the identification of these transcription factors during development, the identification of cell adhesion and guidance cue molecules and the molecular mechanisms underlying the establishment of sensory afferent innervation patterns in the spinal cord are still poorly understood.

Mouse DRG neurons arise from a lineage of migrating neural crest cells (NCCs) expressing *Sox10* [3, 7]. DRG neurons are generated in two overlapping neurogenic waves between embryonic day (E) 9.5 and E13.5, peaking between E10.5 and E11.5, from sensory neuron precursors expressing *Neurogenin 1/2* (*Ngn1/2*) and *Pou4f1* [8–11]. Then, a plethora of subtype-defining transcription factors emerge, and together with extrinsic factors, these factors differentiate sensory neuron precursors into diverse neuron subtypes [12]. Distinct lineages of DRG neurons are defined by the expression of *TrkB* (mechanoreceptors), *TrkC*/*C-Ret* (proprioceptors), or *TrkA* (nociceptors), which constitute approximately 90% of the total DRG neurons [3, 9, 12, 13]. Differentiating DRG primary afferents extend their axons centrally toward the dorsal horn of the spinal cord and peripherally to different targets, such as the skin, muscles, and visceral organs [4]. Although the proper innervation of central and peripheral axons is still poorly understood, a set of cell-adhesion molecules are known to drive these axons to correct synaptic targets.

Mouse dorsal spinal cord neurons arise from progenitor domains located in the ventricular zone of the neural tube in two neurogenic waves [14]. Most superficial dorsal horn neurons are formed in the second neurogenic wave from a pool of progenitors expressing *Gsx1/2* and *Ascl1* [15–18]. The latter wave takes place between E12.5 and E13.5 and gives rise, in a salt and pepper pattern, to two late-born populations, namely dILA and dILB neurons [15, 17]. dILA precursors coexpress *Lbx1*, *Ptf1a*, *Pax2*, and *Lhx1/5* and generate GABAergic neurons [15, 17, 19–21], while dILB precursors coexpress *Lbx1*, *Tlx3*, *Lmx1b*, and *Prrxl1* and produce glutamatergic neurons [22–24]. Both populations migrate dorsally and are responsible for receiving, processing and conveying nociceptive (laminae I-II) and mechanoreceptive (lamina III) information from the periphery to the supraspinal centers [24, 25]. In the adult dorsal horn, both populations have fifteen distinguishable molecular subpopulations, as characterized by single-cell gene expression profiling [26]. Understanding how these various cell types are organized into functional microcircuits, has been the focus of many studies [25, 27].

The paired-like homeodomain protein PRRXL1 is expressed in DRG nociceptive neurons and in their putative targets in the spinal cord dorsal horn and has been shown to be required for the assembly of the DRG-spinal nociceptive circuit [28–30]. *Prrxl1* knockout mice exhibit decreased responsiveness to noxious stimuli that can be associated with developmental defects, namely, misguided innervation of DRG axons into the spinal cord and aberrant migration of spinal dorsal horn neurons followed by increased cell death of both dorsal horn and DRG neurons [23, 28, 31]. Considering these observations, we reasoned that PRRXL1 might regulate the expression of cell adhesion and/or central guidance cue recognition molecules that likely participate in establishing connectivity between DRG nociceptor axon terminals and dorsal horn target neurons.

NEPH1 is a cell-adhesion molecule that belongs to the immunoglobulin superfamily of proteins [32, 33] and is involved in recruiting proteins for actin cytoskeleton polymerization [34, 35]. *Neph1* is highly expressed in the kidney; however, it is also expressed in multiple tissues during animal development, including different regions of the developing nervous system. *Kirre* and *Rst*/*IrreC*, *D. melanogaster* orthologs of *Neph1*, work as guidance molecules in the developing retina and are pivotal in establishing proper connectivity between photoreceptor cells and their targets in the optic ganglia [36, 37]. In addition, the *C. elegans* NEPH1 orthologs SYG-1 and SYG-2 have been identified as key proteins for mediating synaptogenesis and axonal branching through the regulation of subcellular actin cytoskeleton organization [38–41]. However, the role of *Neph1* in the mouse nervous system is still poorly understood. In early developmental stages, *Neph1* is detected in the mesencephalon, optic vesicle, and branchial arches. After neurogenesis, the expression of *Neph1* becomes wider, as detected in the neocortex, hippocampus, cerebellum, DRGs and spinal cord [42, 43]. In adult mice, the NEPH1 protein is expressed in the dendritic shafts of pyramidal neurons and in both pre and postsynaptic neurons in the hippocampus, suggesting a role in synaptogenesis [42]. Although *Neph1* has been found to be expressed in DRGs and the spinal cord [42, 43], spatiotemporal expression pattern analysis and information about the upstream transcriptional regulators of *Neph1* have not yet been reported. Here, we characterized *Neph1* expression in these structures during embryonic development. We also showed that *Neph1* expression is directly regulated by PRRXL1 in the embryonic dorsal spinal cord, but not in the DRGs, and that *Neph1* is necessary for normal neurite arbor morphology.

## Materials and methods

### Animals and tissue preparation

The animals used in this study were maintained in accordance with the European Community Council Directive for the Care and Use of Laboratory Animals of 22 September 2010 (2010/63/EU), National Decreto-Lei 113/2013 and Portaria n. ° 278/2022, and the animal procedures were approved by the i3S Animal Ethics and Portuguese Government Veterinary Committees. NMRI, C57BL6, and CD-1 wild-type mouse strains and the *Neph1* knockout mouse strain [32, 44] were bred and housed at the i3S animal facility, under temperature- and light-controlled conditions. Embryonic day 0.5 (E0.5) was the midpoint of the vaginal plug. Pregnant females were euthanized by cervical dislocation and embryos were collected at different developmental stages. Postnatal animals were euthanized by cervical dislocation and DRGs and spinal cords were collected. For histological analysis, the collected embryos and tissues were immersed in 4% paraformaldehyde (PFA) (Sigma, 30525-89-4) in phosphate buffered saline (PBS) for 48–72 h, cryoprotected in a step gradient of 10%, 20% and 30% sucrose (Fischer Chemical) in PBS until the tissue sank and was embedded in OCT compound (Kaltek). All tissues were sectioned at 12 μm on a cryostat (Leica) and collected on HistoBond + adhesive microscope slides (Marienfeld). For RT-qPCR, the tissues were dissected, roughly lysed in TRIzol (Thermo Fisher Scientific), frozen in liquid nitrogen, and stored at -80 °C. For Western blotting, dorsal spinal cords and kidneys were dissected, frozen in liquid nitrogen, and stored at -80 °C.

### In situ hybridization and immunofluorescence

An RNA probe was produced by in vitro transcription using T3 RNA polymerase (Thermo Fisher Scientific) from a plasmid containing a 537 bp fragment of mouse *Neph1* mRNA [32]. The frozen sections were air-dried, washed with PBS for 15 min at room temperature (RT), and then treated with 0.5 µg/ml proteinase K (PK; Roche) in PBS for 5 min at RT for better probe tissue penetration. PK was inactivated with 1 M glycine (Sigma) for 10 min, after which the tissue sections were postfixed in 4% PFA in PBS for 15 min. Then, the sections were acetylated with acetylation buffer (100 mM triethanolamine-HCl, pH 8 and 0.25% acetic anhydride (Sigma)) for 15 min at RT, prehybridized with prewarmed hybridization buffer (50% formamide (Acros Organics), 2x saline sodium citrate (SSC) buffer, 50 µg/mL yeast RNA (Sigma), 1x Denhardt´s solution (Sigma) and 1 mg/mL salmon sperm DNA (Sigma)) for 1 h at 70 °C and hybridized with prewarmed hybridization buffer containing 1 µg/mL of a *Neph1* digoxigenin (DIG)–labeled RNA probe overnight at 65 °C. Afterwards, the sections were first washed with prewarmed washing buffer (20% formamide, 0.8x SSC, and 1% Tween-20) for 2 h at 70 °C and then washed with Tris-buffered saline (TBS) supplemented with 0.1% Tween 20 (TBS-T), followed by incubation with blocking buffer (10% sheep inactivated serum in TBS-T) and incubation with an anti-DIG antibody coupled to alkaline phosphate (1:2000; Roche, 11093274910) in blocking buffer overnight at 4 °C. To obtain a colorimetric signal, sections were immersed in 1 mg/mL nitro blue tetrazolium (NBT) and 1 mg/mL 5-bromo-4-chloro-3-indolyl phosphate (BCIP) dissolved in alkaline phosphatase buffer (100 mM Tris-HCl, pH 9.5; 100 mM NaCl; 50 mM MgCl_2_; 0.1% Tween-20) and incubated at 4 °C in the dark until the desired signal was obtained. For double or triple staining, in situ hybridization for *Neph1* was followed by immunofluorescence staining for the proteins LMX1B, PAX2, or PRRXL1. Briefly, tissue sections were incubated for two days at 4 °C with the following primary antibodies: guinea pig anti-LMX1B (1:100; gift from Thomas Müller, Max-Delbrück Molecular Medicine Center, Germany), rabbit anti-PAX2 (1:100; Thermo Fisher Scientific, 71-6000), and rabbit anti-PRRXL1 (1:50; [29]). For immunofluorescence staining of TRKA and TRKC, 12 μm frozen tissue sections were air dried, washed with PBS and permeabilized for 3 min with PBS supplemented with 1% Triton X-100. Then, slides were incubated with blocking buffer (TBS supplemented with 10% FBS and 0.1% Triton X-100) for 1 h at RT. Next, the slides were incubated for two days at 4 °C with the following primary antibodies: rabbit anti-TRKA antibody (1:100, Abcam, ab8871) and guinea-pig anti-TRKC antibody (1:100, R&D Biosystems, AF1404) in blocking buffer. A fluorescent signal was obtained by incubation with the secondary antibodies anti-rabbit Alexa Fluor 488 (1:1000; Thermo Fisher Scientific; A21206), anti-guinea-pig Alexa Fluor 568 (1:1000; Thermo Fisher Scientific; A11075) or anti-goat Alexa Fluor 594 (1:1000; Thermo Fisher Scientific; A11058) for 1 h at RT. Fluorescent and bright-field z-stack images were captured and processed as previously described [45].

To quantify the colocalization between *Neph1* with neuron subtype markers, we analyzed three to five lateral dorsal horn areas of transverse sections at thoracic and lumbar axial levels from two mice. Given the limitation of not using DAPI staining in the experiment, *Neph1*^*+*^ cells that did not coexpress with a transcription factor protein (nuclear staining) were counted when in situ hybridization signal (cytosolic staining) was completely, or at least almost completely, surrounding the cell nucleus. For each section analyzed, the number of immunostained cells were manually counted in the magenta channel and double-stained cells in merged channels, using ImageJ open-source software (https://imagej.net/). Results were shown as mean percentage ± standard deviation (SD).

To quantify TRKA and TRKC immunostaining signals, maximum projection of the z-stack images of the dorsal horn were analyzed using ImageJ open-source software. An area covering the ingrowth of sensory fibers into the dorsal horn was selected to measure the signal intensity relatively to the total selected area, as a readout of the density of central sensory innervation. Results were shown as mean percentage ± SD of 2 to 4 images from 2 animals.

### RNA extraction and real-time quantitative PCR

Total RNA was extracted from E14.5 and E16.5 mouse DRGs and dorsal spinal cords and real-time quantitative PCR (qPCR) was performed as previously described [46]. The following sets of primers were designed using Primer3 software (www.primer3plus.com): TGTTACCTGTGGGCATCATT and CTCAACGTCACATCCTTTCG for *Neph1* and GTAATGATCAGTCAACGGGGGAC and CCAGCAAGCTTGCAACCTTAACCA for *hypoxanthine guanine phosphoribosyl transferase* (*Hprt*). The expression of the *Neph1* transcript was normalized to that of the endogenous control gene encoding *Hprt*. The molecular weights of the PCR amplicons were verified by agarose gel electrophoresis. The results are shown as the mean of triplicates ± standard deviation (SD) of two independent experiments.

### Western blotting

Dorsal spinal cords from E14.5 and kidneys from E16.5 embryos were resuspended at a ratio of 1:3 (w (mg)/v (µL)) in lysis buffer (20 mM Tris-HCl pH 7.5, 150 mM NaCl, 1 mM Na_2_EDTA, 1 mM EGTA, and 1% Triton supplemented with phosphatase and protease inhibitor cocktail (NZYTech, MB38301)) and homogenized for 1 cycle of 10 s at 6500 rpm on a MagNa Lyser Instrument (Roche). All samples were sonicated using a Bioruptor UCD-200 (Diagenode) at high power settings for 10 cycles (30 s on/30 seconds off) and the sample concentration was determined by reference to standard concentrations of bovine serum albumin (BSA) using the Bradford method (Bio-Rad Reagent). After quantification, 10 µg of total protein samples were separated via 10% SDS-PAGE and transferred to a nitrocellulose membrane (Bio-Rad) overnight at 4 °C. Then, the blots were blocked with 10% chick albumin dissolved in TBS-T at RT and incubated with the following primary antibodies: rabbit anti-NEPH1 (1:500; [44]) and mouse anti-tubulin (1:40000; Diagenode, AB1157911), overnight at 4 °C. Afterwards, the immunoblots were incubated with AffiniPur donkey anti-rabbit IgG or anti-mouse IgG (1:10,000; Jackson ImmunoResearch, 711-005-152 and 715-035-150, respectively) for 1 h at RT and incubated with Clarity Western ECL Substrate (Bio-Rad). Chemiluminescence signals were captured and quantified using ChemiDocTM XRS system (Bio-Rad). The expression of NEPH1 protein was normalized to that of the housekeeping protein tubulin.

### Chromatin immunoprecipitation

PRRXL1 chromatin immunoprecipitation (ChIP) assays were performed essentially as previously described [46], with the following minor modification: the chromatin fixation time with di(N-succinimidyl) glutarate was 20 min. To assess ChIP enrichment, we performed quantitative PCR (qPCR). For that purpose, we designed primers targeting PRRXL1-bound regions at the *Neph1* locus using Primer3 software (www.primer3plus.com). The primers used to target the genomic regions downstream of the *Neph1* transcription start site (TSS) were TGTGATGAGGGTTTTGATGG and CTCTTCTCCCGTTTCTCCTG for (+ 87,222), GAATTGGATTGCGGATTTCT and AGGATGCTAATGCCCACAC for (+ 76,001), AAGTGTGCTGGGTGGTAGC and TCTGAGTAGCCCTAGCTGTCC for (+ 54,020), AGGGGACCTGAAACACAGAG and AGGCTTCTCCTCCCTTCC for (+ 34,266) and GCCACTTCGCTTTGATGATA and ATGGCTCTCCTGAAGCATTT for (+ 13,570). The primer sets used for *Prrxl1* genomic coordinates were reported elsewhere [47]. To retrieve the PRRXL1 binding profile at the *Neph1* locus, the PRRXL1 ChIP-seq dataset was uploaded to the UCSC Genome Browser (https://genome-euro.ucsc.edu/) [48] and displayed together with the ENCODE ChIP-seq datasets [49] for histone 3 lysine 4 monomethylation (H3K4me1), H3K4 trimethylation (H3K4me3), and H3K27 acetylation (H3K27ac) from E14.5 mouse neural tube chromatin and conservation across 30 vertebrate species.

### Statistical analysis

For in situ hybridization, the number of mouse embryos ranged from 2 to 5, the exact number for each experiment is shown in the figure legends. In the ChIP-qPCR assays, the results are plotted as a percentage of input chromatin yield from ChIP with or without the anti-PRRXL1 antibody and as the mean of triplicates ± standard deviation (SD) of two independent experiments. Statistical significance was assessed by Student’s *t* test. The mouse samples used were from *Prrxl1* knockouts (*n* = 3 at E14.5 and *n* = 5 at E16.5) and wild-types (*n* = 3 at E14.5 and *n* = 5 at E16.5) for qPCR, from *Prrxl1* knockouts (*n* = 4) and wild-types (*n* = 6) for Western blotting, and from *Prrxl1* knockouts (*n* = 2) and wild-types (*n* = 2) for TRKA and TRKC immunofluorescence. Genotype group values, expressed as the mean ± SD, were compared by Student’s *t*-test, and *P* values < 0.05 were considered significant.

### Primary culture of DRG neurons and immunofluorescence

DRGs from E18.5 wild-type (*n* = 4) or *Neph1* knockout (*n* = 3) mouse embryos were dissected and placed in neurobasal medium supplemented with 10% fetal bovine serum (FBS), 1% GlutaMAX and 1% penicillin/streptomycin (Thermo Fisher Scientific). The tissues were subjected to enzymatic digestion with 0.125% collagenase IV-S (Sigma Aldrich) for 2 h at 37 °C, followed by mechanical dissociation with glass Pasteur pipettes until no cluster of cells was visible. The cells were purified by decantation and resuspended in neurobasal medium supplemented with 1% penicillin/streptomycin, 2% B27 (Thermo Fischer Scientific) and 50 ng/mL of NGF (Promega). Cells were plated on poly-L-lysine (Quimigen) and Laminin (Sigma Aldrich) -coated glass coverslips and maintained at 37 °C in a humidified 5% CO2 atmosphere for 20 h. Coverslips were washed with ice-cold PBS and fixed with 4% PFA for 15 min. Cells were then blocked with 5% FBS in PBS-T for 1 h and incubated overnight with rabbit anti-βIII-tubulin (1:1000; Synaptic Systems, 302302) in 5% FBS in PBS-T at 4 °C. After washing with PBS-T, the cells were incubated for 1 h at RT with donkey anti-rabbit Alexa Fluor 568 (1:1000; Thermo Fisher Scientific; A11042) in 5% FBS in PBS-T. Coverslips were mounted on glass slides with Fluoroshield mounting media (Sigma-Aldrich) for image acquisition. Fluorescence images of single neurons were captured on a Zeiss Axio Imager Z1 microscope with an EC-Plan-Neofluar 20x/0.50 Ph2 objective.

### Assessment of neurite number using semiautomated Sholl analysis and statistics

The morphological data of the neurons were quantified using the Bonfire Program [50] according to the developer’s instructions using MATLAB R2018b (MathWorks). Neurons with no neurite growth (total neurite length < 20 µM) or neurons where the segmentation of neurites was not possible due to overlapping with neurites from other neurons were excluded from the analysis. Neurite segmentation and processing were performed by an experimenter blinded to the genotype condition. Statistical analysis was performed using Prism (GraphPad v10.1.2). For Sholl analyses, statistical significance was evaluated by the multiple Mann–Whitney test, which compares ranks with multiple comparisons adjustment for the false discovery rate (FDR) using the two-stage setup method of Benjamini, Krieger, and Yekutieli. For the analysis of dendrite branching, terminal points, number and length, statistical significance was assessed by the Mann–Whitney U test. The level of significance was set at *p* < 0.05 and *q* < 0.05. The data are presented as described in the figure legends.

## Results

### Characterization of *Neph1*-expressing neurons in the developing DRGs and spinal cord

**Fig. 1:**
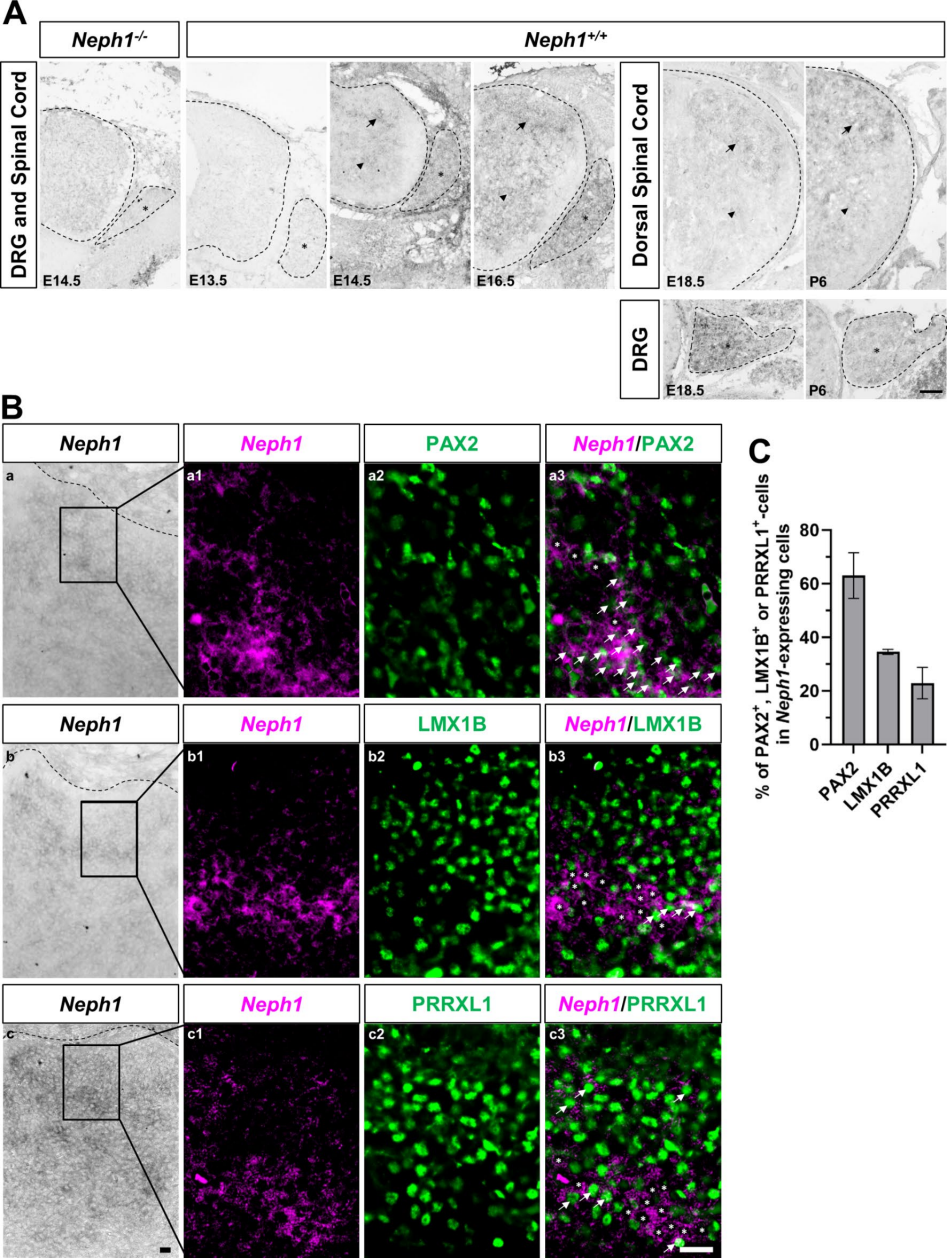
*Neph1* is expressed in developing DRGs and spinal cord. **A** Spatiotemporal analysis of *Neph1* mRNA expression in DRGs and spinal cord by in situ hybridization. In the mouse DRGs (asterisk), *Neph1* expression was broadly observed from E14.5 onward. In the spinal cord, *Neph1* expression initiated at E14.5, is detected in the dorsal horn (arrow) and the ventral spinal cord (arrowhead), and expands to more superficial layers of the dorsal horn from E16.5 onward. As a negative control, we performed in situ hybridization at E14.5 *Neph1* knockout embryos and no staining was detected. Representative transverse sections through the lumbar spinal cord of E13.5 (*n* = 4), E14.5 (*n* = 5), E16.5 (*n* = 4), E18.5 (*n* = 3), P6 (*n* = 3) wild-type and E14.5 *Neph1* knockout embryos (*n* = 2) are shown. Scale bar: 100 μm. **B** *Neph1* is mainly expressed in GABAergic neurons in the spinal cord dorsal horn. (a-c) In situ hybridization of transverse sections of the spinal cords of E14.5 wild-type embryos. (a1-c1) Bright-field in situ hybridization signals of *Neph1* mRNA were converted into magenta pseudocolour. (a2-c2) Immunostaining of either PAX2, LMX1B, or PRRXL1 (a3, b3, and c3 in green, respectively) protein. (a3-c3) Double staining of *Neph1* mRNA (in magenta) with either PAX2, LMX1B or PRRXL1 (a3, b3, and c3 in green, respectively) protein. Note the extensive colocalization of *Neph1* with PAX2 (a1, arrows), but less co-localization with LMX1B (b1, arrows) and PRRXL1 (c1, arrows), or the absence of *Neph1* colocalization with these markers (asterisks). **C** Quantitative analysis showed coexpression of transcription factors PAX2 (using three tissue sections (*n* = 3) from 2 embryos), LMX1B (*n* = 3, 2 embryos) or PRRXL1 (*n* = 5, 2 embryos) with *Neph1*. The *Neph1*^+^ cell counts were as follows: PAX2 (41 ± 16), LMX1B (38 ± 6) or PRRXL1 (47 ± 16). The data are shown as mean percentage ± SD of the cells that express each transcription factor analyzed in the *Neph1*^+^ cell population in the lateral dorsal horn at E14.5. Scale bar: 20 μm

To characterize the expression pattern of *Neph1*, we performed in situ hybridization using a specific mRNA probe for *Neph1* at different embryonic stages. In the DRGs, soon after the neurogenic waves, *Neph1* was broadly expressed from E14.5 to postnatal day 6 (P6) and was almost undetected at E13.5 (Fig. 1A, asterisk). Likewise, *Neph1* expression was not detected in a single cell transcriptomic study from E9.5 to E13.5 developing spinal cords [51]. As expected, *Neph1* expression was not detected in E14.5 *Neph1* knockouts, confirming the specificity of the mRNA probe. In the spinal cord, *Neph1* was also expressed after neurogenic waves, starting at E14.5 in the deep dorsal horn (Fig. 1A, arrows) and in the ventral spinal cord (Fig. 1A, arrowheads), after which expands to the most superficial layers of the dorsal horn from E16.5 onward (Fig. 1A, arrows). Notably, publicly available mouse in situ hybridization data [52] showed that *Neph1* was broadly expressed in the DRGs at postnatal day 4 (P4), while in the dorsal horn, it was expressed in superficial laminae I-III throughout postnatal life (at P4 and P56), therefore maintaining the same expression pattern that we observed during late embryonic development.

To better characterize the neuronal populations that express *Neph1* in the developing dorsal spinal cord, we performed in situ hybridization for *Neph1* followed by immunofluorescence for major neuron subtype markers at E14.5. Since *Neph1* expression initiates at E14.5 in the dorsal horn of the spinal cord, we reasoned that these neurons are originated during the second wave of neurogenesis. To determine whether *Neph1* is expressed in dILA (GABAergic) or dILB (glutamatergic) neuronal populations, we analyzed the coexpression of markers of both populations. We observed that *Neph1* was largely coexpressed with PAX2, a dILA marker (Fig. 1B a-a3, arrows and Fig. 1C), while the coexpression of *Neph1* with either LMX1B or PRRXL1, which are dILB markers, was much less pronounced (Fig. 1B b-c3, arrows and Fig. 1C).

### *Neph1* is regulated by PRRXL1 in the embryonic dorsal spinal cord

**Fig. 2:**
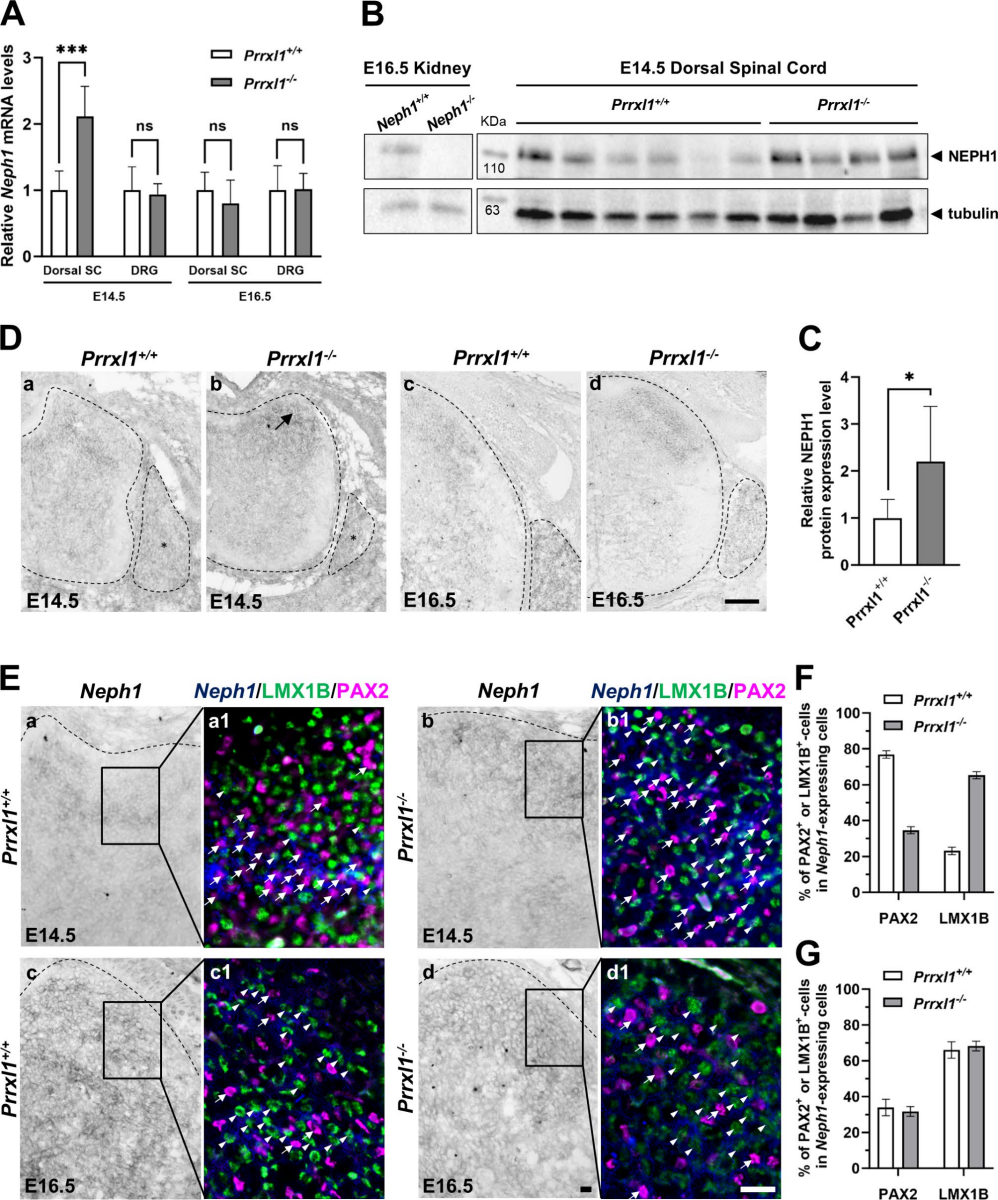
PRRXL1 prevents early expansion of the *Neph1* expression domain in superficial dorsal horn. **A** Reverse transcription followed by qPCR was performed using RNA extracted from either the dorsal spinal cord or DRG tissues of wild-type (*Prrxl1*^*+/+*^, *n* = 3) and *Prrxl1* knockout (*Prrxl1*^*−/−*^, *n* = 3) E14.5 embryos, being n a pool of three embryos. For E16.5 embryos, dorsal spinal cord or DRG tissues from wild-type (*Prrxl1*^*+/+*^, *n* = 5) and *Prrxl1* knockout (*Prrxl1*^*−/−*^, *n* = 5) were used. *Neph1* expression was normalized with *Hprt* housekeeping gene. ***P < 2.00E-05, compared to the wild-type group. *Neph1* expression is upregulated in the dorsal spinal cord of *Prrxl1* knockout embryos at E14.5, but not at E16.5. **B** and **C** NEPH1 protein expression levels were assessed by Western blotting using dorsal spinal cord tissue from wild-type (*n* = 6) and *Prrxl1* knockout (*n* = 4) E14.5 embryos. NEPH1 protein expression was normalized with tubulin housekeeping protein. *P < 2.00E-02. *Neph1* wild-type and knockout kidney samples were used to validate the specificity of the anti-NEPH1 antibody. NEPH1 protein expression is increased in the dorsal spinal cord of *Prrxl1* knockout embryos. **D** In situ hybridization analysis of *Neph1* expression. Representative transverse sections through the spinal cord of E14.5 (a and b) and E16.5 (c and d) embryos of the wild-type (a, *n* = 5; c, *n* = 3) and *Prrxl1* knockout (b, *n* = 5; d, *n* = 3) genotypes. The arrow indicates ectopic expression of *Neph1* in the superficial dorsal horn. Scale bar: 100 μm. **E** Expression of LMX1B or PAX2 in *Neph1*-expressing neurons. (a-d) In situ hybridization of *Neph1* in transverse sections of the lumbar spinal cords of E14.5 (a, b) and E16.5 (c, d) embryos from the wild-type (a, c) and *Prrxl1* knockout (b, d) genotypes. (a1-d1) Triple staining of LMX1B protein (green) and PAX2 protein (red) with *Neph1* mRNA (blue). Bright-field in situ hybridization signals were converted into blue pseudocolour signals. Ectopic expression of *Neph1* in the superficial dorsal horn of *Prrxl1* knockout E14.5 embryos mainly occured in LMX1B-positive cells (b1, arrowheads). The arrows indicate *Neph1*^+^/PAX2^+^ cells, while the arrowheads label *Neph1*^+^/LMX1B^+^ cells. **F** Quantitative analysis showed that a large number of prospective glutamatergic neurons of the dorsal horn switch on *Neph1* expression in the absence of PRRXL1 at E14.5. Coexpression of transcription factors PAX2 or LMX1B with *Neph1* (from *Prrxl1*^*+/+*^ mice, using four tissue sections (*n* = 4) from 2 embryos; from *Prrxl1*^*−/−*^ mice, *n* = 3, 2 embryos). The *Neph1*^+^ cell counts were as follows: *Prrxl1*^*+/+*^ mice (62 ± 21) and *Prrxl1*^*−/−*^ mice (150 ± 22). **G** Quantitative analysis showed no alteration in the *Neph1* expression between prospective glutamatergic and GABAergic neurons in the absence of PRRXL1 at E16.5. Coexpression of transcription factors PAX2 or LMX1B with *Neph1* (from *Prrxl1*^*+/+*^ mice, *n* = 3, 2 embryos; from *Prrxl1*^*−/−*^ mice, *n* = 5, 2 embryos). The *Neph1*^+^ cell counts were as follows: *Prrxl1*^*+/+*^ (108 ± 21) and *Prrxl1*^*−/−*^ (103 ± 8). **F** and **G** The data are shown as mean percentage ± SD of the cells that express each transcription factor analyzed in the *Neph1*^+^ cell population in the lateral dorsal horn from wild-type and *Prrxl1* knockout embryos at E14.5 and E16.5. Scale bar: 20 μm

*Prrxl1* knockout embryos exhibit defects on the ingrowth of TRKA afferent fibers into the spinal cord as well as abnormal migration of dorsal horn neurons [28, 53]. One possible explanation is that these developmental defects result from altered expression of guidance cues, their receptors, and/or cell-adhesion molecules. To obtain new insight into this topic, we used a microarray expression profiling dataset, previously generated by us from embryonic DRGs and the dorsal spinal cord of wild-type and *Prrxl1* knockout mice (F. A. Monteiro, unpublished data). Strikingly, gene expression microarray data indicated that the expression of a gene encoding cell-adhesion transmembrane protein *Neph1* was upregulated in the dorsal spinal cord of *Prrxl1* knockout embryos at E14.5 (fold change (fc) = 1.32, *p* = 5.84E-06) as compared to wild-type embryos, but no significant change was detected in the DRGs (fc = 0.98, *p* = 6.27E-01). Likewise, an independent quantitative real-time PCR experiment confirmed the upregulation of *Neph1* mRNA expression in the dorsal spinal cord of *Prrxl1* knockout embryos at E14.5 (fc = 2.11, *p* < 2.00E-05), whereas no changes were observed in E14.5 DRGs (fc = 0.94, *p* < 9.00E-01) (Fig. 2A). No statistically significant changes were detected in the dorsal spinal cord (fc = 0.79, *p* < 1.00E-01) or DRGs (fc = 1.01, *p* < 9.08E-01) between *Prrxl1* knockout and wild-type embryos at E16.5 (Fig. 2A). In line with these results, the NEPH1 protein level was also increased in the dorsal spinal cord of *Prrxl1* knockout embryos at E14.5 as assessed by Western Blot (fc = 2.20, *p* < 2.00E-02) (Fig. 2B-C). Notably, the band detected for NEPH1 was absent in the kidney protein extract of the *Neph1* knockout as compared with the wild-type embryos, thus validating the specificity of the anti-NEPH1 antibody. By in situ hybridization, we observed ectopic expression of *Neph1* in the most superficial laminae of the dorsal horn of *Prrxl1* knockout embryos at the lumbar, thoracic and cervical (Fig. 2D a-b and S1 a-d, arrows) axial levels, although no differences were observed in the DRGs of *Prrxl1* knockout embryos (Fig. 2D a-b, asterisks). In situ hybridization confirmed that *Neph1* expression did not change in the dorsal spinal cord and DRGs of *Prrxl1* knockout as compared with wild-type embryos at E16.5 (Fig. 2D c-d).

To evaluate whether cells ectopically expressing *Neph1* in *Prrxl1* knockouts were from the dILB (glutamatergic) or dILA (GABAergic) lineage, we performed in situ hybridization for *Neph1* followed by immunofluorescence for LMX1B or PAX2, respectively, in the dorsal spinal cords of *Prrxl1* knockout and wild-type mice at E14.5. In addition to being a marker of glutamatergic dILB cells, we also used *Lmx1b* as a surrogate marker for *Prrxl1*-positive cells because LMX1B is extensively coexpresses with PRRXL1 in superficial dorsal horn neurons and because *Lmx1b* expression is not affected in *Prrxl1* knockouts at E14.5 [23]. In the wild-type embryos, the expression of *Neph1* was mainly associated with PAX2-positive cells and less extensively associated with LMX1B-positive cells (Fig. 2E a-a1 and F, arrows and arrowheads, respectively). However, in the *Prrxl1* knockout embryos, ectopic expression of *Neph1* in most superficial layers was extensively present in the LMX1B-positive glutamatergic cells (Fig. 2E b-b1 and F, arrowheads). These findings suggest that a large number of prospective glutamatergic neurons in the dorsal horn switch on *Neph1* expression in the absence of PRRXL1, indicating that PRRXL1 was necessary to suppress, in glutamatergic cells, the expression of *Neph1*. At E16.5, however, *Neph1* colocalized with PAX2 but more extensively with LMX1B in the dorsal horn of *Prrxl1* knockout and wild-type mice (Fig. 2E c-d1 and G, arrows and arrowheads, respectively), indicating that the repression exerted by PRRXL1 on *Neph1* expression in the superficial laminae of the dorsal horn was restricted to E14.5, which is in line with the findings of the *Neph1* mRNA expression analysis (Fig. 2A).

**Fig. 3:**
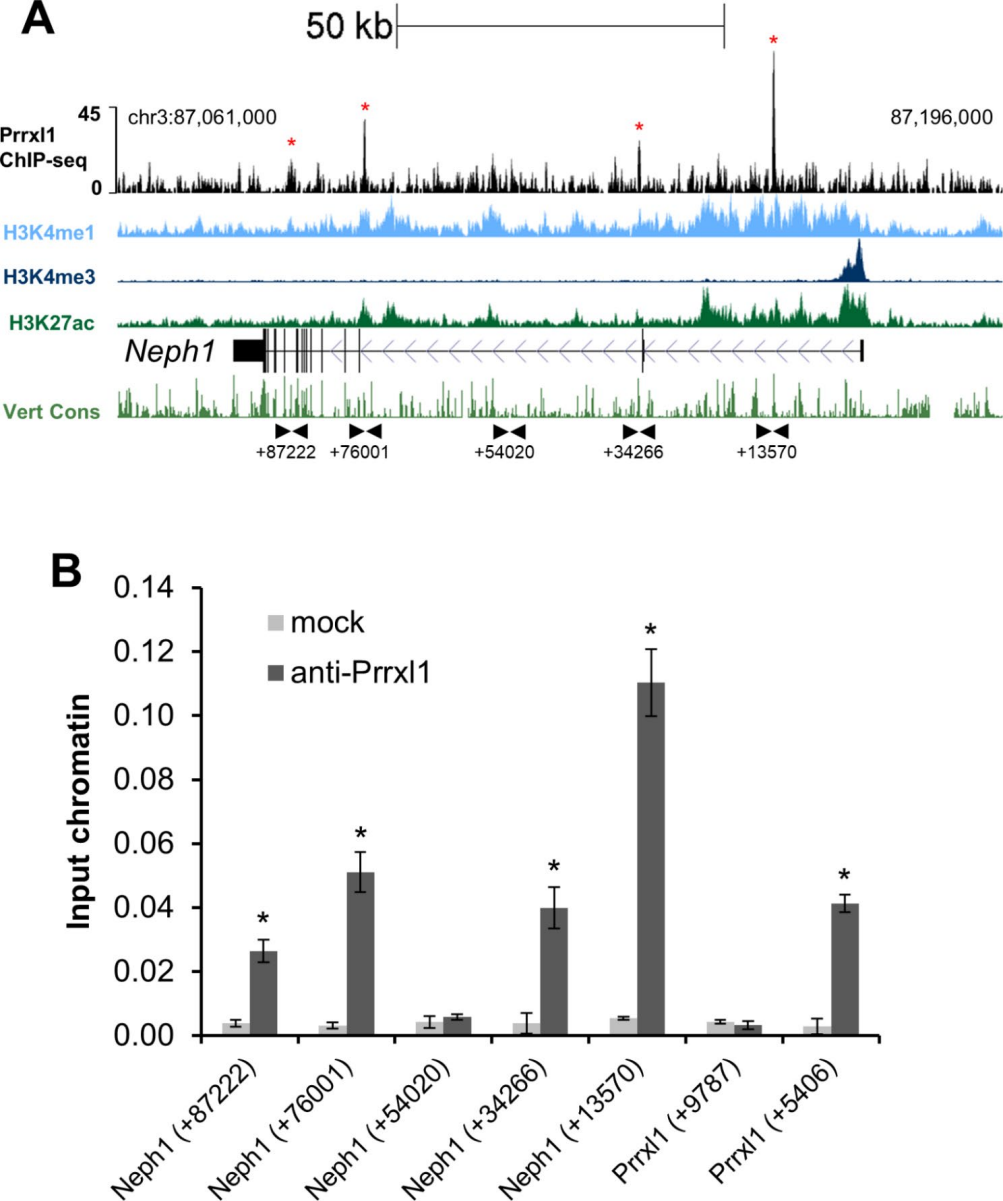
PRRXL1 binds to the *Neph1* locus in embryonic dorsal spinal cord chromatin. **A** PRRXL1 binding enrichment (in black) at different sites in the *Neph1* locus. Dorsal spinal cord chromatin from E14.5 embryos was immunoprecipitated with an anti-PRRXL1 antibody and subjected to next-generation sequencing (ChIP-Seq). H3K4me1 (light blue), H3K4me3 (dark blue), K3K27ac (dark green), the *Neph1* genomic structure and direction of transcription (black), and multispecies vertebrate conservation (light green) plots are shown. The ENCODE annotations are from histone marks of ChIP-Seq datasets generated using the E14.5 mouse neural tube [49]. The data tracks were retrieved using the UCSC genome browser with the GRCm38/mm10 assembly [48]. The arrowheads represent the positions of the primers in the base pairs (+ 13,570, + 34,266, + 54,020, +76,001 and + 87,222) relative to the transcription start site of *Neph1*. The binding sites validated by ChIP-qPCR are marked by red asterisks. **B** PRRXL1 binds to multiple sites at the intronic regions of *Neph1* locus (sites + 87,222, +76,001, + 34,266, and + 13,570). We used primers spanning a *Prrxl1* exonic region (site + 9787) as a negative control and primers spanning a *Prrxl1* intronic region (site + 5406) as a positive control. Both regions were previously validated [47]. *P < 1.00 E-04, as compared to *Neph1* (site + 54,020)

To determine whether *Neph1* transcriptional expression is regulated by PRRXL1 through direct interaction, we searched for PRRXL1 binding sites in the genomic locus of *Neph1* (Supplementary Table 1), using a PRRXL1 ChIP-seq dataset generated from the E14.5 mouse dorsal spinal cord (F. A. Monteiro, unpublished data). The peak calling algorithm mapped three regions bound by PRRXL1 located at evolutionarily conserved intronic sequences (Fig. 3A). Independent ChIP-qPCR assays confirmed that PRRXL1 was enriched at intronic regions within *Neph1* locus (sites + 13,570, +34,266, + 76,001 and + 87,222) but not at site + 54,020 as expected from the ChIP-seq enrichment profile. The + 9787 and + 5406 sites at the *Prrxl1* locus were used as negative and positive controls, respectively, for PRRXL1 binding (Fig. 3B), as previously reported [47]. It is well established that the enrichment of the histone marks H3K4me1 and H3K4me3, which, in combination with H3K27ac, define active enhancer or promoter regions, respectively [54]. Consistent with the assumption that PRRXL1 binding at multiple sites in the *Neph1* locus entails a regulatory effect, we observed enrichment of the H3K4me1 and H3K27ac histone marks at PRRXL1 peaks, suggesting that PRRXL1 occupies active *cis*-regulatory elements (Fig. 3A). These results indicate that PRRXL1 directly controls *Neph1* expression in glutamatergic neurons in the developing dorsal horn, possibly through multiple regulatory regions.

### *Neph1* is required for the branching of distal neurites

**Fig. 4:**
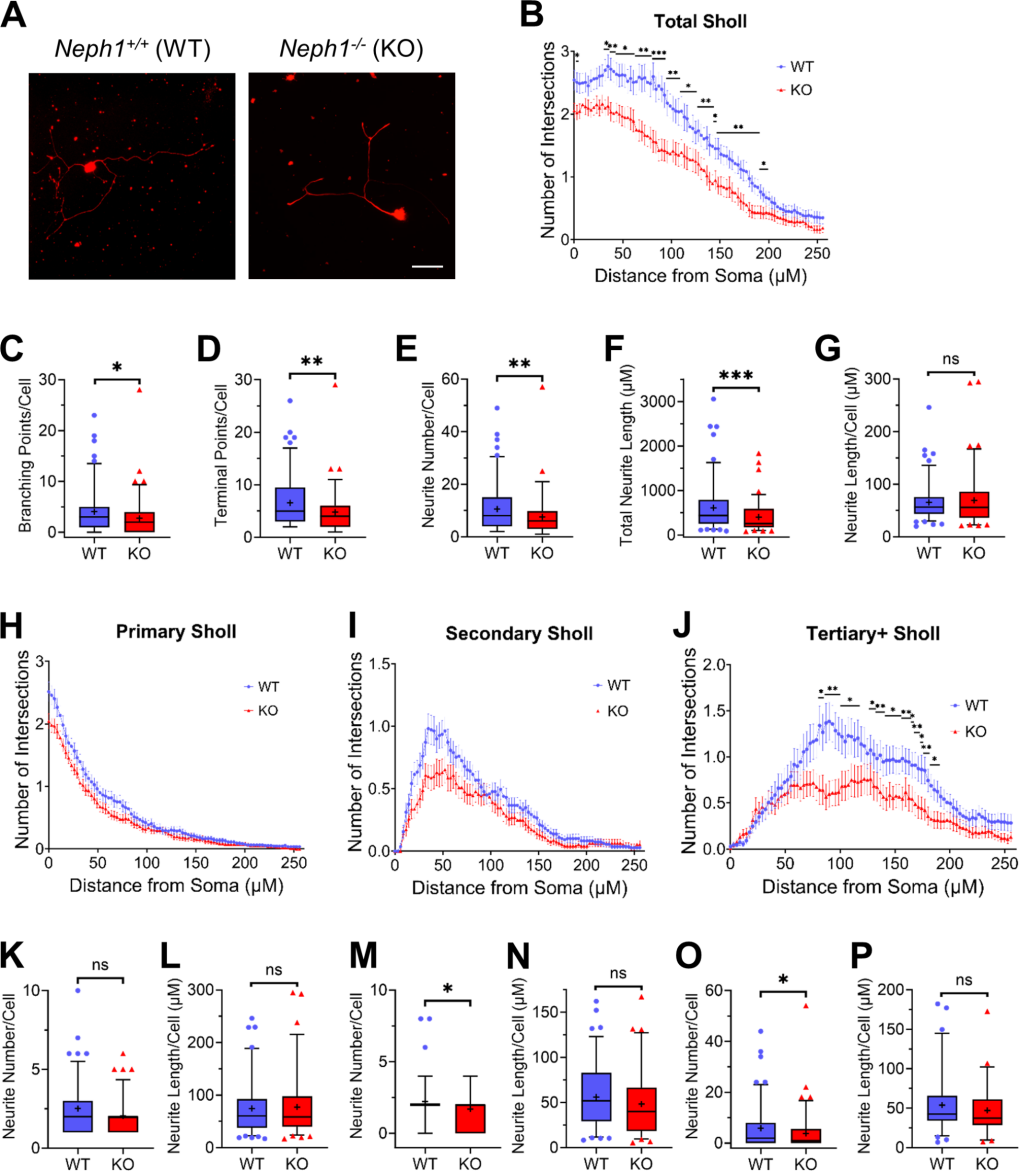
Branching and the number of distal neurites were reduced in DRG primary cultures from *Neph1*^*−/−*^ mice. **A** Representative images of DRG neurons collected from E18.5 wild-type (WT) or *Neph1*^*−/−*^ (KO) mice immunolabeled with βIII-tubulin. Scale bar: 50 μm. **B** Sholl analysis of all orders of branches (Total Sholl) showed that the absence of NEPH1 significantly decreased neurite branching mainly at 32–197 μm from the cell body (*p < 0.05, **p < 0.01, ***p < 0.001 and *q* < 0.05). **C** The average number of branch points per cell was reduced in the absence of NEPH1 (*p = 0.0121). **D** The average number of terminal points per cell was reduced in the absence of NEPH1 (**p = 0.0039). **E** Total number of neurites per cell is reduced in the absence of NEPH1 (**p = 0.0040). **F** The total neurite length per cell was reduced in the absence of NEPH1 (***p = 0.0003). **G** The average neurite length per cell was not significantly different in the absence of NEPH1 (^ns^*p* = 0.6664). **H**–**J** Sholl analysis using the inside-out (conventional) labeling method. Sholl analyses of primary neurites (Primary Sholl) **(H)** and secondary neurites (Secondary Sholl) **(I)** did not show statistically significant changes. **J** Sholl analysis of tertiary and higher order neurites (Tertiary + Sholl) showed that the absence of NEPH1 significantly decreased neurite branching at 81–116 μm and at 128–189 μm from the soma (*p < 0.05; ** *p* < 0.01 and *q* < 0.05). **K**–**P** Number **(K**,** M**,** O)** and length **(L**,** N**,** P)** of neurites per cell divided into orders using the inside-out (conventional) labeling method. The absence of NEPH1 did not significantly change the number of first order neurites **(K)** (^ns^*p* = 0.0576), however the number of second order **(M)** and third or higher order neurites **(O)** was significantly reduced (*p = 0.0112 and *p = 0.0244, respectively). The absence of NEPH1 did not significantly change the length of the first **(L)** (^ns^*p* = 0.9376), second **(N)** (^ns^*p* = 0.0973) or third or higher order neurites **(P)** (^ns^*p* = 0.1434). In **B** and **H**–**J**, the data are shown as the mean ± SEM, and the statistical analysis was performed with multiple Mann–Whitney tests, multiple comparisons were performed by the false discovery rate (method by Benjamini, Krieger, and Yekutieli). The level of significance for both the *p* and *q* values was set to < 0.05. In **C**–**G** and **K**–**P**, the data are shown in box-and-whisker plots, where the boxes span 50% of the data, whiskers span 90% of the data, the horizontal line within each box represents the median, and the plus represents the mean. Statistics were calculated using a two-tailed Mann-Whitney U test. ns, not significant. *n* = 109 neurons from 5 WT animals and *n* = 92 neurons from 4 KO animals

As *Neph1* is a downstream target of PRRXL1 in the superficial dorsal horn of E14.5 mouse embryos, we hypothesized that the cell-adhesion molecule *Neph1* works as an effector gene of PRRXL1, possibly regulating neuronal differentiation and connectivity. In fact, it was previously shown that *C. elegans* SYG-1 (a NEPH1 ortholog) mediates synaptogenesis and axonal branching [38–41]. To investigate whether *Neph1* plays a similar role in neurite branching in mammals, we cultured low-density cultures of dissociated DRG neurons isolated from either *Neph1* knockout or wild-type mice at E18.5 (Fig. 4A) and measured eventual changes in the neuritic arbor through Sholl analyses [55]. All the branch orders grouped together (Total Sholl) showed significantly reduced branching, mainly at 32–197 μm from the cell body, in cultured DRG neurons from the *Neph1* knockout mice, as compared with wild-type mice (Fig. 4B and Supplementary Table 2). The total number of branching points, terminal points and neurites per cell, as well as the total length of the neurites per cell, were significantly diminished in cultured DRG neurons from *Neph1* knockout mice (Fig. 4C-F and Supplementary Tables 3, 4, 5). However, the average size of individual neurites did not significantly change between the genotypes (Fig. 4G and Supplementary Table 5), suggesting that NEPH1 regulates neurite branching, but not neurite growth.

To analyze in more detail the alterations in the neurite arbor of cultured *Neph1* knockout DRG neurons, we performed conventional inside-out Sholl analysis [56]. This method separately analyzes the neurites that extend from the cell body (primary neurites), those that stem from primary neurites (secondary neurites), and those that stem from secondary neurites and so on (tertiary and higher order neurites). We found that although primary and secondary neurites did not significantly change the neurite branching pattern (Fig. 4H, I and Supplementary Table 2), compared with those in the wild-type controls, the tertiary and higher order neurites in cultured DRG neurons of the *Neph1* knockout mice exhibited significantly reduced branching at 81–116 μm and 128–189 μm from the soma (Fig. 4J and Supplementary Table 2). Similarly, we observed that the number of secondary, tertiary and higher order neurites was significantly decreased in the DRG neurons of the *Neph1* knockout mice (Fig. 4M, O and Supplementary Table 4), while there was no significant difference in the number of primary neurites (Fig. 4K and Supplementary Table 4). As shown in Fig. 4G, no significant changes were found in the average length of neurites per cell, independent of the neurite order (Fig. 4L, N, P and Supplementary Table 6). These results indicate that NEPH1 is specifically required for the branching of distal neurites (tertiary and higher order neurites), thus regulating neurite number, while neurite extension is not affected.

## Discussion

In this study, we characterized the expression pattern of *Neph1* in mouse embryonic and early postnatal DRGs and the dorsal spinal cord. In the DRGs, *Neph1* is broadly expressed from E14.5 onward, while in the dorsal spinal cord, it is expressed in discrete domains within dIL neurons. Moreover, we showed that PRRXL1 directly represses *Neph1* expression in glutamatergic neurons of the superficial dorsal horn at E14.5 but has no regulatory effect on the DRGs neither in both tissues at E16.5. We also showed by Sholl analyses that *Neph1* is necessary for proper neuritic arbor morphology by regulating distal branching but not neurite growth. Considering that *Prrxl1* is required for proper assembly of the DRG-spinal cord circuit, our results suggest that *Neph1* acts downstream of *Prrxl1* in this process.

At the end of DRG neurogenesis (∼ E12.5), primary afferents start to project to the dorsal horn of the spinal cord through the dorsal root entry zone, where they innervate specific layers of the spinal cord around E14, after a waiting period [4]. We showed that *Neph1* is broadly expressed in the DRGs from E14.5 until perinatal development, resembling the expression pattern at the E16.5 stage previously described [42]. Besides, *Neph1* is expressed in subtypes of *TrkA*, *TrkB* and *TrkC* sensory neurons at E15.5, as assessed by single cell transcriptomics analyses [11]. Considering the role of invertebrate *Neph1* orthologs in axon guidance, branching, synapse formation and maintenance [37–41], it is tempting to hypothesize that *Neph1* may also intervene in the establishment of connectivity and synaptogenesis between the primary afferent sensory neurons (dedicated to nociception, mechanoreception and proprioception) and their targets in the spinal cord. Here, we demonstrated that *Neph1* expression is not regulated by PRRXL1 in embryonic DRG neurons at E14.5 or E16.5. A search of global gene expression studies of gene knockout DRGs for key regulators of sensory neuron specification, including genes encoding the transcription factors ISL1, POU4F1, RUNX3, and C-MAF [57–59], did not reveal *Neph1* deregulation. Therefore, the upstream regulators of *Neph1* in DRGs remain to be uncovered.

In the developing spinal cord, lamination of the dorsal horn becomes evident from E15.5 onward [53]. In the adult dorsal spinal cord, specific laminae are responsible for processing different types of sensory information; namely, lamina I and II mainly receive thermo, pruri, and nociceptive primary afferent projections, while lamina III mostly receives mechanoreceptive terminals [60–62]. Our results showed that *Neph1* starts being expressed in neurons mainly located in presumptive lamina III at E14.5, and then its expression domain expands to cells located in more superficial laminae of the dorsal horn (presumptive laminae I-II) at E16.5 and is maintained throughout embryonic and postnatal development. In fact, the pattern we obtained at E14.5 is similar to that described elsewhere [43]. We detected *Neph1* expression in both GABAergic dILA and glutamatergic dILB neurons, which is in line with the findings of previous single-cell RNA sequencing studies in which *Neph1* was detected in subsets of GABAergic and glutamatergic dorsal horn neurons [26, 63, 64]. To better understand the neuronal subtypes that express *Neph1*, we mined the harmonized atlas of single-cell RNA sequencing data collected from early postnatal to adult mouse spinal cords [65]. *Neph1* was expressed in six excitatory and ten inhibitory cell subtypes (Figure S2). Four *Neph1*^*+*^ dorsal excitatory subtypes (i.e. Excit-1, -8, -11 and − 19) mainly coexpressed with *Lmx1b* as compared with *Prrxl1*, while nine *Neph1*^*+*^ dorsal inhibitory subtypes (i.e. Inibit-1 to -6 and Inibit-9 to -11) coexpressed with *Pax2* (Figure S3). We also showed that *Neph1* expression was markedly increased in the superficial dorsal horn of *Prrxl1* knockout mice at E14.5, suggesting that its expression is negatively regulated by PRRXL1. Indeed, these genes were barely coexpressed at E14.5. This ectopic expression of *Neph1* resembled the expression pattern observed in wild-type mice at E16.5 and was extensively observed in LMX1B^+^-neurons (Fig. 2E, F), which highly coexpressed with PRRXL1 in the wild-type mice [23]. This finding led to the hypothesis that PRRXL1 regulates the expression of *Neph1* in a time-specific manner, which was validated since *Neph1* is highly coexpresses with LMX1B at E16.5 and no differences were observed in the *Prrxl1* knockout mice at this embryonic stage. Our findings suggest that PRRXL1 acts as a negative transcriptional regulator of *Neph1* in the embryonic spinal cord, preventing premature expression in superficial laminae I-II of the dorsal horn. This regulatory mechanism is most likely mediated by direct interactions with multiple evolutionarily conserved intronic regions within the *Neph1* locus. In addition, the enrichment of histone marks at PRRXL1-bound sites suggested that PRRXL1 binds to active distal *cis*-regulatory elements (i.e., enhancers). As *Neph1* is a downstream effector gene of *Prrxl1* in a large subset of glutamatergic dorsal horn neurons in most superficial layers, it is probable that the function of *Neph1* is related to *Prrxl1*. In fact, *Prrxl1* is required for proper central innervation of nociceptive DRG axons to target laminae I-II and for the migration of dorsal horn neurons [28, 53], while *Neph1* has been associated with neuronal connectivity [38–42, 66, 67]. In fact, other studies have shown spatiotemporally restricted expression of SYG-1 (a NEPH1 ortholog in *C. elegans*) in layers, a pattern also observed for other cell-adhesion molecules [68], which are thought to be critical for differential cell adhesion governing the sites of neurite formation and synaptic specificity [69]. Given that *Neph2* and *Neph3* (*Neph1* paralogs) were not differentially expressed in the *Prrxl1* knockouts (fc = 1.01, *p* = 9.04E-01 for *Neph2* and fc = 1.02, *p* = 6.23E-01 for *Neph3*), it is conceivable that *Neph1* might, at least, mediate some of the functions of *Prrxl1* in DRG-spinal circuitry formation.

In the nervous system, *Neph1* homologs have been shown to mediate synaptogenesis in *C. elegans* [38–41] and in the *Drosophila* visual system [37, 70]. Despite the fact that *Neph* genes (*Neph1*, *2* and *3*) are widely expressed in the nervous system in vertebrates [42, 43], our knowledge of these genes is limited. Only *Neph2* and *Neph3* have been implicated in synaptogenesis and axon coalescence in the mouse olfactory bulb [71–73]. In mice around E14, DRG neuronal central projections start to innervate the developing dorsal spinal cord in a lamina-specific innervation pattern, however information about which cell membrane molecules control the laminar position of sensory neuron subtype target fields is scarce [12, 25]. Neuronal branching and synapse formation are tightly linked to developmental events during the assembly of synaptic circuits [74]. Both processes depend on local actin polymerization dynamics [63] and are mediated by transmembrane cell-adhesion molecules, such as NEPH1 [38, 39]. We hypothesize that in *Prrxl1* knockouts, the abnormal increase in *Neph1* expression in superficial dorsal horn neurons at this developmental stage may lead to the dysregulation of dendritic branching and, consequently, the alteration of synaptic contacts and neuronal networks.

We found no apparent alterations in the expression levels of TRKA and TRKC in the dorsal horn of *Neph1* knockout embryos (Figure S4), most likely due to lack of enough resolution to measure the density of sensory innervation. Thus, we used primary cultures of mouse DRG neurons as a paradigm to test the hypothesis that mouse *Neph1* participates in the formation of neuritic arbors, a process that is highly regulated by intrinsic and extrinsic factors and is essential for proper functioning of the nervous system [75, 76]. The three *Neph* variants are expressed in DRG neurons in partially overlapping patterns [42, 43]. Strikingly, we found a decrease in the number of distal neurites from the soma of cultured DRG neurons from *Neph1* knockout mice. This finding suggests that NEPH2, NEPH3, and eventually other cell adhesion molecules cannot compensate for the absence of NEPH1 [41]. The lack of compensation could be explained by differences in the function of the NEPH1 paralogs or a reduction in the amount of NEPH proteins at distal neurites below critical levels to perform branching. Interestingly, the absence of NEPH1 did not impact on neurite length, suggesting that elongation of neurites is a NEPH1-independent process. This assumption is further reinforced by the fact that NEPH1 is involved in the regulation of local actin dynamics [34, 35] and that the inhibition of actin polymerization blocks axon branching without affecting axon growth [77].

Although the role of *Neph1* has been well described in renal physiology [32–34], its function in the mammalian nervous system has only started to be addressed [71–73]. Our study provides a detailed characterization of *Neph1* in the DRGs and the dorsal spinal cord during mouse embryogenesis and the early postnatal stage, paving the way for future functional studies, and identifies PRRXL1 as the first upstream regulator of *Neph1*, a candidate effector gene for DRG axons to target dorsal horn neurons. To determine the role of *Neph1* in establishing functional connectivity between the DRG and dorsal spinal cord and since *Neph1* knockout mice die shortly after birth from proteinuria [32], a conditional knockout mouse strategy, in which *Neph1* is specifically ablated in either the DRGs or dorsal spinal cord, would be appropriate.

### Electronic supplementary material

Below is the link to the electronic supplementary material.

**Supplementary Material 1: Table 1.** Genomic coordinates of PRRXL1 binding sites annotated to Neph1/Kirrel determined by ChIP-Seq

**Supplementary Material 2: Table 2.** Sholl analysis of all orders (total Sholl) (**sheet 1**), first order (primary Sholl) (**sheet 2**), second order (secondary Sholl) (**sheet 3**), and third and higher order (tertiary+ Sholl) (**sheet 4**) neurites from DRG neurons from *Neph1* knockout and wild-type E18.5 embryos

**Supplementary Material 3: Table 3.** Number of branch (**sheet 1**) and terminal points (**sheet 2**) per DRG neuron from *Neph1* knockout and wild-type E18.5 embryos

**Supplementary Material 4: Table 4.** Numbers of total, primary, secondary, and tertiary and high order neurites per DRG neuron from *Neph1* knockout and wild-type E18.5 embryos

**Supplementary Material 5: Table 5.** Total and average neurite length per DRG neuron from *Neph1* knockout and wild-type E18.5 embryos

**Supplementary Material 6: Table 6.** Average length of primary (**sheet 1**), secondary (**sheet 2**) and tertiary and high order neurites (**sheet 3**) per DRG neuron from *Neph1* knockout and wild-type E18.5 embryos

**Supplementary Material 7: Figure S1 (related to Figure 2D).** ***Neph1*** **expression is increased in the superficial dorsal horn of** ***Prrxl1*** **knockout embryos at the thoracic and cervical axial levels.** In situ hybridization analysis of *Neph1* expression. Representative transverse sections through the spinal cord of E14.5 embryos at cervical (a and b) and thoracic (c and d) axial levels from wild-type (**a**, n = 3; **c**, n = 5) and *Prrxl1* knockout (**b**, n = 5; **d**, n = 5) genotypes. The arrow indicates ectopic expression of *Neph1* in the superficial dorsal horn. Scale bar: 100 μm

**Supplementary Material 8: Figure S2. Analysis of** ***Neph1/Kirrel*** **expression across mouse spinal cord subtypes.** Unsupervised single-nuclei RNA-seq clustering after dimensionality reduction and cell neighborhood identification allowed the detection of *Neph1/Kirrel* in excitatory and inhibitory subtypes of the spinal cord. Graphs were obtained from data mining the website https://seqseek.ninds.nih.gov/

**Supplementary Material 9: Figure S3. Analysis of gene expression across mouse spinal cord subtypes using a set of genes.** *Neph1/Kirrel* coexpressed with *Lmx1b* and in a lesser extent with *Prrxl1* in excitatory subtypes Excit-1, Excit-8, Excit-11 and Excit-19, which are located in the dorsal spinal cord; however, *Neph1/Kirrel* did not coexpress either with *Lmx1b* or *Prrxl1* in excitatory cell types Excit-28 and Excit-37, which are located in the center and ventral spinal cord, respectively. *Neph1/Kirrel* coexpressed with *Pax2* in inhibitory subtypes Inhib-1 to -6, Inhib-9 to -11, which are located in the dorsal spinal cord, and Inhib-16, which is located in the mid spinal cord. Green rectangles indicate excitatory cell types and red rectangles indicate inhibitory cell types. Data mining was obtained from https://seqseek.ninds.nih.gov/ website

**Supplementary Material 10: Figure S4 (related to Figure 4). TRKA and TRKC afferent projections were not altered in the dorsal horn of** ***Neph1*** **knockout embryos.** (**A**) Immunofluorescence staining of TRKA in the dorsal horn from wild-type and *Neph1* knockout embryos at E14.5. Arrow indicates the expression of TRKA. Scale bar: 50 μm. (**B**) Quantitative analysis showed no alteration in the expression levels of TRKA in the dorsal horn of *Neph1* knockout (using four tissue sections (n = 4) from 2 animals) as compared to wild-type (n = 4, 2 animals) embryos at E14.5 (^ns^*p* = 0.1425). (**C**) Immunofluorescence staining of TRKC in the dorsal horn from wild-type and *Neph1* knockout embryos at E14.5. Arrow indicates the expression of TRKC. Scale bar: 50 μm. (**D**) Quantitative analysis showed no alteration in the expression levels of TRKC in the dorsal horn of *Neph1* knockout (n = 3, 2 animals) as compared to wild-type (n = 4, 2 animals) embryos at E14.5 (^ns^*p* = 0.4206). (**B, D**) Relative expression levels of TRKA (**B**) or TRKC (**D**) were normalized by the total area used in the quantification and data are shown as the mean of relative signal intensity ± SD, and the statistical analysis was performed with unpaired *t*-test

## Data Availability

Data supporting the findings of this manuscript is either provided within the manuscript or supplementary information files or will be provided upon request.

## Abbreviations

BCIP: 5-bromo-4-chloro-3-indolyl phosphate
BSA: Bovine serum albumin
ChIP: Chromatin immunoprecipitation
DREZ: Dorsal root entry zone
DRGs: Dorsal root ganglia
E: Embryonic day
NCCs: Neural crest cells
Ngn1/2: Neurogenin 1/2
NBT: Nitro blue tetrazolium
PFA: Paraformaldehyde
PBS: Phosphate buffered saline
qPCR: Real-time quantitative PCR
RT: Room temperature
SSC: Saline sodium citrate
TBS: Tris-buffered saline
