# Supplementary figures and images for: Neph1 is required for neurite branching and is negatively regulated by the PRRXL1 homeodomain factor in the developing spinal cord dorsal horn
Source: Neural Dev. 2024 Jul 24;19:13. doi: 10.1186/s13064-024-00190-6 (PMC11271021; doi:10.1186/s13064-024-00190-6)

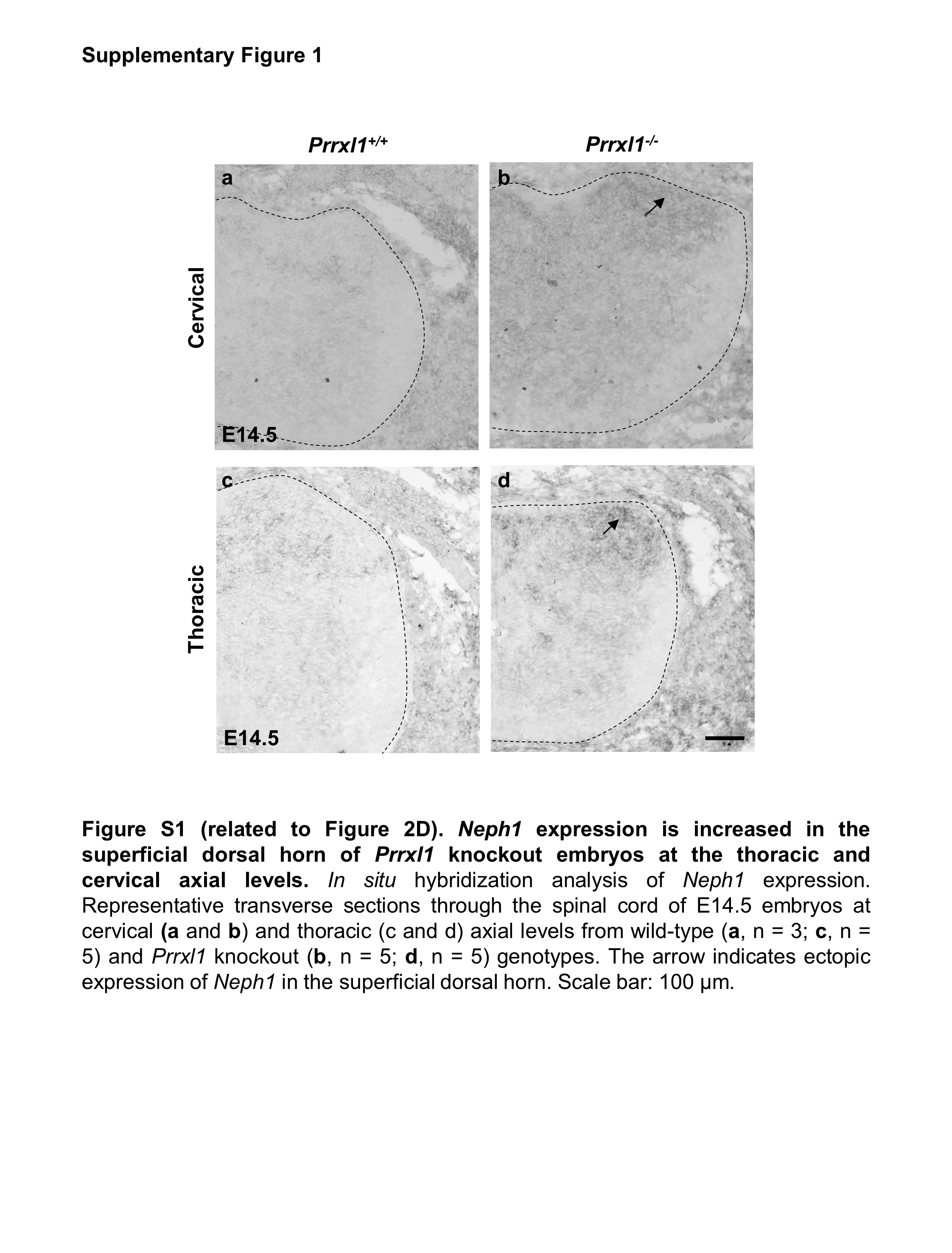

Supplement: Supplementary file 7 — Supplementary Material 7: Figure S1 (related to Figure 2D). Neph1 expression is increased in the superficial dorsal horn of Prrxl1 knockout embryos at the thoracic and cervical axial levels. In situ hybridization analysis of Neph1 expression. Representative transverse sections through the spinal cord of E14.5 embryos at cervical (a and b) and thoracic (c and d) axial levels from wild-type (a, n = 3; c, n = 5) and Prrxl1 knockout (b, n = 5; d, n = 5) genotypes. The arrow indicates ectopic expression of Neph1 in the superficial dorsal horn. Scale bar: 100 μm [file 13064_2024_190_MOESM7_ESM.tif]

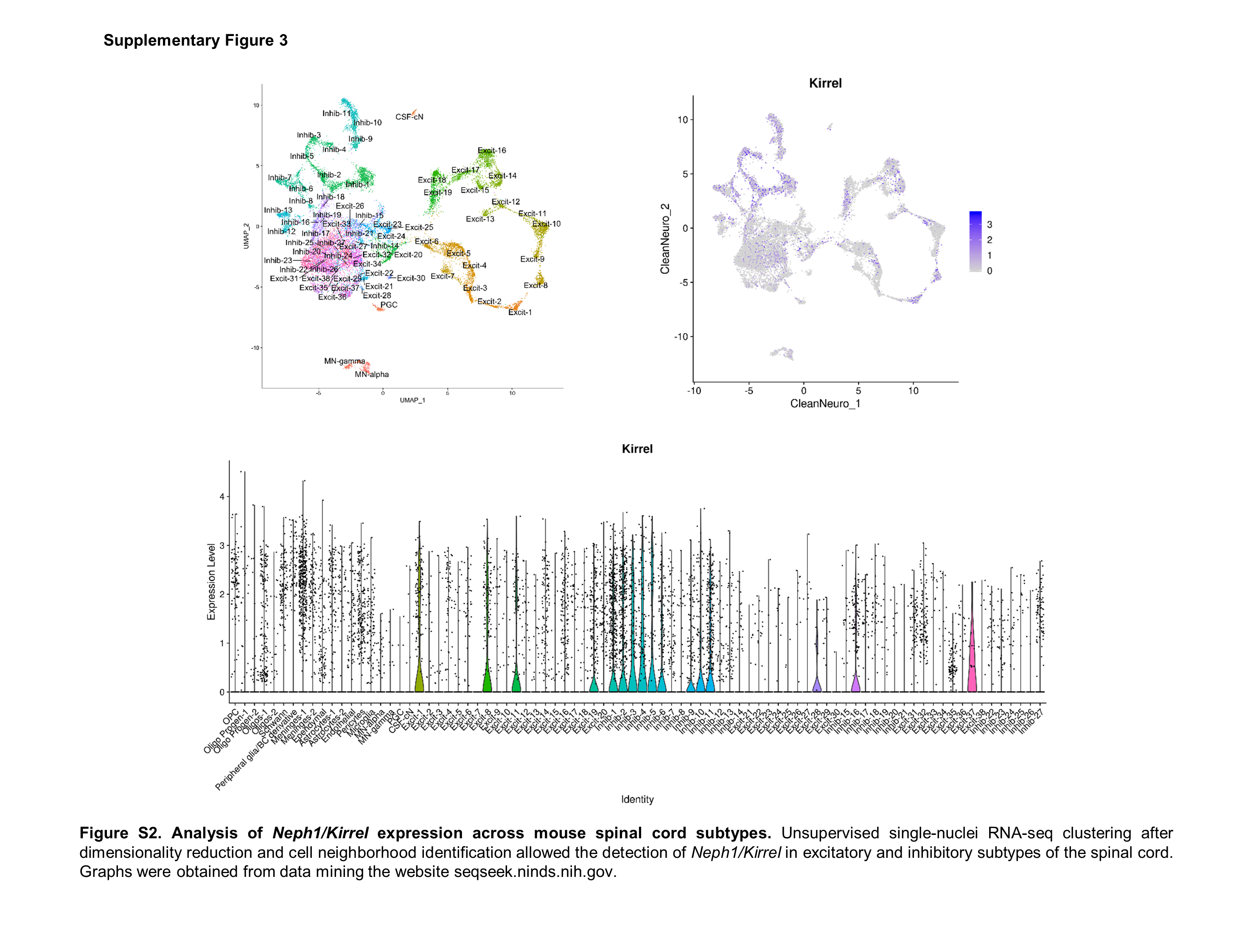

Supplement: Supplementary file 8 — Supplementary Material 8: Figure S2. Analysis of Neph1/Kirrel expression across mouse spinal cord subtypes. Unsupervised single-nuclei RNA-seq clustering after dimensionality reduction and cell neighborhood identification allowed the detection of Neph1/Kirrel in excitatory and inhibitory subtypes of the spinal cord. Graphs were obtained from data mining the website https://seqseek.ninds.nih.gov/ [file 13064_2024_190_MOESM8_ESM.tif]

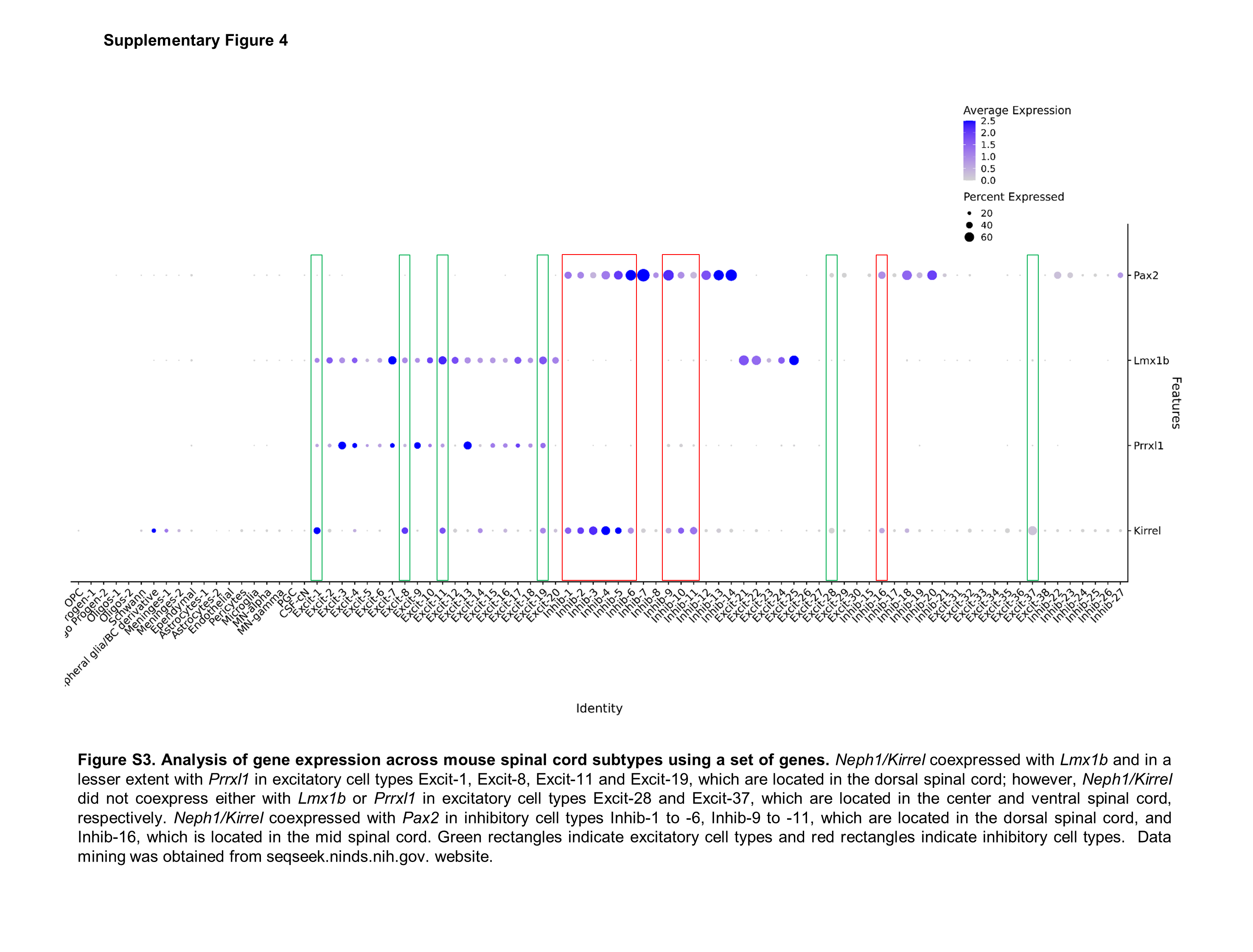

Supplement: Supplementary file 9 — Supplementary Material 9: Figure S3. Analysis of gene expression across mouse spinal cord subtypes using a set of genes. Neph1/Kirrel coexpressed with Lmx1b and in a lesser extent with Prrxl1 in excitatory subtypes Excit-1, Excit-8, Excit-11 and Excit-19, which are located in the dorsal spinal cord; however, Neph1/Kirrel did not coexpress either with Lmx1b or Prrxl1 in excitatory cell types Excit-28 and Excit-37, which are located in the center and ventral spinal cord, respectively. Neph1/Kirrel coexpressed with Pax2 in inhibitory subtypes Inhib-1 to -6, Inhib-9 to -11, which are located in the dorsal spinal cord, and Inhib-16, which is located in the mid spinal cord. Green rectangles indicate excitatory cell types and red rectangles indicate inhibitory cell types. Data mining was obtained from https://seqseek.ninds.nih.gov/ website [file 13064_2024_190_MOESM9_ESM.tif]

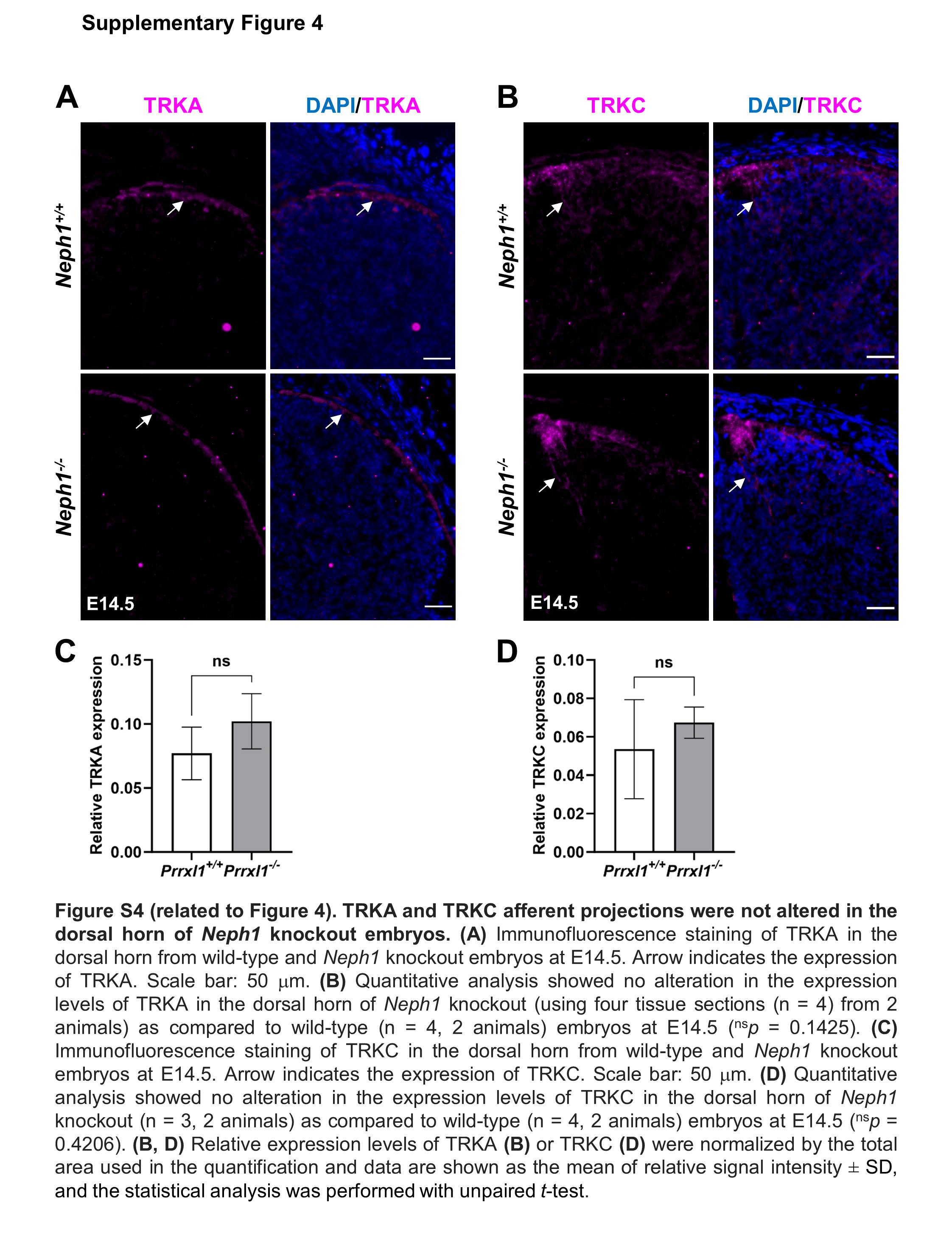

Supplement: Supplementary file 10 — Supplementary Material 10: Figure S4 (related to Figure 4). TRKA and TRKC afferent projections were not altered in the dorsal horn of Neph1 knockout embryos. (A) Immunofluorescence staining of TRKA in the dorsal horn from wild-type and Neph1 knockout embryos at E14.5. Arrow indicates the expression of TRKA. Scale bar: 50 μm. (B) Quantitative analysis showed no alteration in the expression levels of TRKA in the dorsal horn of Neph1 knockout (using four tissue sections (n = 4) from 2 animals) as compared to wild-type (n = 4, 2 animals) embryos at E14.5 (nsp = 0.1425). (C) Immunofluorescence staining of TRKC in the dorsal horn from wild-type and Neph1 knockout embryos at E14.5. Arrow indicates the expression of TRKC. Scale bar: 50 μm. (D) Quantitative analysis showed no alteration in the expression levels of TRKC in the dorsal horn of Neph1 knockout (n = 3, 2 animals) as compared to wild-type (n = 4, 2 animals) embryos at E14.5 (nsp = 0.4206). (B, D) Relative expression levels of TRKA (B) or TRKC (D) were normalized by the total area used in the quantification and data are shown as the mean of relative signal intensity ± SD, and the statistical analysis was performed with unpaired t-test [file 13064_2024_190_MOESM10_ESM.tif]
